# Glutathione Peroxidase (GPx) and Superoxide Dismutase (SOD) in Oropharyngeal Cancer Associated with EBV and HPV Coinfection

**DOI:** 10.3390/v12091008

**Published:** 2020-09-09

**Authors:** Małgorzata Strycharz-Dudziak, Sylwia Fołtyn, Jakub Dworzański, Małgorzata Kiełczykowska, Maria Malm, Bartłomiej Drop, Małgorzata Polz-Dacewicz

**Affiliations:** 1 Chair and Department of Conservative Dentistry with Endodontics, Medical University of Lublin, 20-059 Lublin, Poland; 2 Department of Otolaryngology, Masovian Specialist Hospital, 26-617 Radom, Poland; sp.foltyn@interia.pl; 3 Department of Internal Diseases, Masovian Specialist Hospital, 26-617 Radom, Poland; kdworzanski@onet.eu; 4 Department of Medical Chemistry, Medical University of Lublin, 20-059 Lublin, Poland; malgorzata.kielczykowska@umlub.pl; 5 Department of Information Technology and Medical Statistics, Medical University of Lublin, 20-059 Lublin, Poland; maria.malm@umlub.pl (M.M.); bartlomiej.drop@umlub.pl (B.D.); 6 Department of Virology with SARS Laboratory, Medical University of Lublin, 20-059 Lublin, Poland; m.polz@umlub.pl

**Keywords:** oropharyngeal cancer, coinfection, EBV, HPV, oxidative stress, GPx, SOD

## Abstract

Recent reports have pointed to the link between persistent inflammation, oxidative stress, and carcinogenesis; however most of the studies concerning the role of viruses in head and neck cancer (HNC) are focused mainly on one type of virus. Our present study aimed to study the relationship between Epstein–Barr virus/human papilloma virus (EBV/HPV) coinfection and glutathione peroxidase (GPx) and superoxide dismutase (SOD) level in oropharyngeal cancer. Fresh-frozen tumor tissue samples were collected from 128 patients with oropharyngeal cancer infected with EBV or HPV or with EBV/HPV coinfection. After DNA extraction, EBV and HPV DNA was detected using a polymerase chain reaction (PCR) assay. GPx and SOD activity was determined in homogenates of cancer tissue using diagnostic kits produced by Randox Laboratories. Both GPx and SOD activity was statistically lower in patients with EBV/HPV coinfection than in a single EBV or HPV infection. Analysis of GPx and SOD activity in relation to histological grading and tumor, node (TN) classification revealed that in poorly-differentiated tumors, the level of antioxidant enzymes was lower compared with well-differentiated lesions and in cases with greater tumor dimensions and lymph-node involvement, both GPx and SOD activity was decreased. Further studies are necessary to clarify the influence of interplay between EBV, HPV, and oxidative stress on malignant transformation of upper aerodigestive tract epithelial cells.

## 1. Introduction

Oncogenic virus-induced cancers are an important global concern nowadays. According to various reports, although all human cancers are non-communicable diseases, infections may contribute to about 13–20 % of global human cancer burden, and it is estimated that about 35 % of all infection-associated malignancies are attributable to human papilloma virus (HPV) and Epstein–Barr virus (EBV) [1,2,3,4]. EBV and HPV infections are associated with different cancers, including gastric, cervical, and breast carcinoma, as well as head and neck cancer (HNC) [5]. As some studies revealed, EBV and HPV coinfection plays an important role in the malignant transformation of epithelial cells [6,7].

EBV establishes a latent infection, reactivated periodically into the lytic cycle, which plays an important role in the pathogenesis of tumors related to EBV [8]. During latent infection, several specific viral proteins, such as Epstein–Barr nuclear antigen 1 (EBNA1), Epstein–Barr virus-encoded RNA 1 and 2 (EBER1 and EBER2), and BamHI-A rightward transcripts (BART) as well as latent membrane protein 1 and 2 (LMP1, LMP2) are expressed [9]. LMP1 is regarded as the most important oncoprotein encoded by EBV, which can trigger multiple signaling cascades, such as nuclear factor-kappa B (NF-κB), MAPK, JNK/AP1, PI3K, and as a result it may generate various cancer hallmarks. EBNA1 expressed in all types of EBV latency is the only protein expressed in all EBV-positive tumors and sometimes the only protein expressed. It is responsible for maintaining the EBV genome in latently infected cells, but it can also contribute to cell immortalization and malignant transformation via interferences with tumor suppressors, induction of DNA damage, and altering of signaling pathways [10].

HPV oncogenic potential is connected with E6 and E7 oncoprotein expression, whereas the oncogenic properties of EBV are attributed to the expression of latent genes, including LMP1 and LMP2 [6,11]. HPV E6 protein inactivates tumor suppressor p53-mediated DNA damage and the apoptosis pathway, while HPV E7 protein inactivates the tumor suppressor pRb-mediated cell cycle regulation pathway [12].

Oxidative stress is defined as the imbalance between the production of reactive oxygen species (ROS) and antioxidant defense, which can lead to damage of various cell components and influence cellular processes, such as proliferation or apoptosis, involved in the development of cancer [13]. ROS act as second messengers in many intracellular signaling pathways responsible for maintaining homeostasis of the cell with its immediate environment. Moreover, ROS take part in various cellular functions, such as intracellular signal transduction, proliferation, cell apoptosis, regulation of gene expression, and participate in the process of protein phosphorylation. They may also induce transcription factors (e.g., NF-_K_B), which causes disorders in the normal functioning of cells involved in the development of cancer [13,14].

The most important defense against oxidative damage is antioxidants. The main enzymes with antioxidant activity are glutathione peroxidase (GPx), superoxide dismutase (SOD), and catalase (CAT). Superoxide dismutase is the most important antioxidant enzyme in aerobic cells, responsible for the elimination of superoxide radicals. SOD catalyzes the dismutation of hydrogen peroxide and molecular oxygen. Glutathione peroxidase (GPx) in cells is the main hydrogen peroxide-scavenging enzyme which converts this molecule to water. SOD and GPx can directly counterbalance the oxidant attack and protect the cells against DNA damage [14].

Numerous studies have shown that both EBV and HPV infection are associated with the production of reactive oxygen species (ROS) and may activate signaling pathways mediated by oxidative stress, thus influencing migration of inflammatory cells and leading to mutations and tissue damage [15,16,17].

Recent reports pointed to the link between persistent inflammation, oxidative stress, and carcinogenic processes [1,18,19,20]. Therefore, our present research focuses on the analysis between coinfection and oxidative stress parameters in oropharyngeal cancer. The aim of the study was the assessment of GPx and SOD activity in patients with oropharyngeal carcinoma with EBV/HPV coinfection in comparison to oropharyngeal cancer patients with a single HPV or EBV infection.

## 2. Materials and Methods

### 2.1. Patients

The present study involved 128 patients with a diagnosed and histopathologically confirmed oropharyngeal squamous cell carcinoma (SCC), who were also infected with EBV or HPV, or coinfected with both EBV and HPV. In this group, 54 patients were EBV(+), 44 patients were HPV(+), and 30 patients were coinfected with EBV and HPV. The patients were hospitalized at the Otolaryngology Division of the Masovian Specialist Hospital in Radom, Poland. The patients had not received radiotherapy or chemotherapy before. Clinical and epidemiological characteristics of the patients are presented in Table 1. The study groups did not differ in terms of epidemiological and clinical features.

The tissue samples were collected from all patients during surgery and frozen at −80 °C until analysis. Tumor, node, metastasis (TNM) classification was determined during primary diagnosis according to the criteria of the Union for International Cancer Control (UICC) [21]. Histological grading was performed according to World Health Organization criteria, which divide tumors into three types: well differentiated (G1), moderately differentiated (G2), and poorly differentiated (G3) [22].

The research was approved by the Medical University of Lublin Ethics Committee and was in accordance with good clinical practice (GCP) regulations (No. KE-0254/135/2017, 25.05.2017).

### 2.2. DNA Extraction from Fresh Frozen Tumor Tissue

Fragments of the fresh frozen tumor tissue (20 mg) collected from the patients with oral squamous cell carcinoma (OSCC) were cut and homogenized in a manual homogenizer Omni TH/Omni International/Kennesewa/Georgia/USA. DNA was extracted using a protocol as described in the DNeasy Tissue Kit Handbook (QiagenGmBH, Hilden, Germany). Purified DNA was quantified by spectrophotometry (Epoch Microplate Spectrophotometer, BioTek Instruments Inc., Vinooski, Vermont, USA). Isolated DNA was kept at −20 °C until the test was conducted. To verify the quality of the obtained DNA (presence of inhibitors of Polymerase Chain Reaction), a β-globin assay was performed.

### 2.3. Detection of EBV DNA

EBV DNA detection: All PCR reactions were carried out in the final volume of 25 μL using HotStartTaq DNA Polymerase (Qiagen, Hilden, Germany). Concentrations of PCR reaction components were prepared as follows: 2.0 mM MgCl_2_, 0.2 mMdNTPs, 0.5 μM of each forward and reverse primers and 0.5 U of HotStartTaq polymerase. During each run, the samples were tested together with one negative (nuclease-free water) and one positive control (EBV-positive cell line, Namalwa, ATCC-CRL-1432).

### 2.4. HPV Detection and Genotyping

It was performed using the INNO-LiPA HPV Genotyping Extra assay (Innogenetics, Gent, Belgium). The kit is based on the amplification of a 65 bp fragment from the L1 region of the HPV genome with SPF10 primer set. PCR products are subsequently typed with the reverse hybridization assay. This kit identifies 28 HPV genotypes: HPV 6, 11, 16, 18, 26, 31, 33, 35, 39, 40, 43, 44, 45, 52, 52, 53, 54, 56, 58, 59, 66, 68, 69, 70,71, 73, 74, and 82.

### 2.5. Oxidant Parameters

The tissue samples were rinsed with 0.9 % NaCl and stored at −80 °C until the analysis. Tissue homogenates (10 % *w/v*) were prepared in 0.1 mol. l–1Tris-HCl buffer, pH = 7.4 using a laboratory MPW-120 homogenizer, and supernatants were obtained by centrifugation at 5000× *g* for 30 min.

The following oxidant parameters were determined in homogenates of cancer tissue: activities of superoxide dismutase (SOD) and glutathione peroxidase (GPx).

SOD activity was determined using diagnostic kit RANSOD produced by RANDOX (Randox Laboratories Ltd., Crumlin, County Antrim, UK) according to Arthur and Boyne [23] and expressed in U of SOD/10 mg of protein.

GPx activity was determined using diagnostic kit RANSEL produced by RANDOX (Randox Laboratories Ltd., Crumlin, County Antrim, UK) according to Paglia and Valentine [24] and expressed in U of GPx/mg of protein. Protein was measured using the Bradford method [25]. The assays were performed with the use of spectrophotometer SPECORD M40 (Carl Zeiss, Jena, Germany).

### 2.6. Statistical Analysis

Descriptive statistics were used to present patient baseline characteristics. Means and standard deviations (SD) were calculated. For variables with non-normal distribution, the Mann–Whitney *U*-test and Kruskal–Wallis test were used. Pearson’s chi-square test was used to investigate the relationship between clinical and demographical parameters. Statistical significance was defined as *p*  <  0.05.

## 3. Results

Both GPx and SOD values were the highest in HPV(+) patients, lower in EBV(+), and the lowest in EBV/HPV coinfection (GPx level in HPV(+) = 4.99 U/mg of protein, in EBV(+) = 4.47 U/mg of protein, in EBV/HPV coinfection = 3.75 U/mg of protein; SOD level in HPV(+) = 11.49 U/100 mg of protein, in EBV(+) = 11.06 U/100 mg of protein, in EBV/HPV coinfection = 9.77 U/100 mg of protein). There was no significant difference in the activity of GPx and SOD in patients infected with only EBV or HPV. However, the activity of both antioxidant enzymes in oropharyngeal cancer tissue in patients with EBV/HPV coinfection was statistically lower than in a single EBV or HPV infection (Table 2).

Comparison between GPx and SOD activity and histological grading as well as TN classification was also carried out (Table 3). In poorly-differentiated tumors (G3), the activity of GPx and SOD was lower compared with well and moderately-differentiated lesions (G1-G2). Similar relationships were stated between tumor dimensions, lymph-node involvement, and the values of oxidative stress parameters. In cases with greater tumor dimensions (T3-T4) and lymph-node involvement (N3-N4), the level of antioxidant enzymes was lowered. Therefore, all analyzed parameters had the lowest values in EBV/HPV coinfected patients with poorly-differentiated tumors (G3) and in stages T3-T4, N3-N4.

## 4. Discussion

Coinfection with two or more oncogenic viruses may induce neoplastic transformation [6,7,26,27]. It is interesting that some malignant lesions develop in a minority of chronically infected persons, usually after many years of persistent infection [1,4,28]. However, most of the studies concerning the role of viruses in HNC development are focused mainly on one type of virus, and only few recent reports analyze the possible relationship and interplay between two different viruses [6,7].

Our previous studies revealed that HPV/EBV coinfection was found in 37.8% of patients with oropharyngeal cancer. Comparison of a single EBV infection and a coinfection, regarding different clinical and pathological features of the lesion, showed a significant relationship between histological grading and presence of EBV/HPV coinfection. In the group with EBV/HPV coinfection, the prevalence of poorly-differentiated tumors (G3) was over four-fold more frequent than in the group with EBV infection alone. [29]. In the next study, decreased activity of antioxidant enzymes (GPx and SOD) in oropharyngeal cancer tissue, particularly in EBV-positive patients was stated. Significantly lower GPx and SOD values were found in patients with reactivation of EBV infection [30].

Jalouli et al. [26] reported occurrence of EBV/HPV coinfection in 15–30 % of patients with oral squamous cell carcinoma in industrialized countries and in 6–65 % of OSCC patients in developing countries. In another study carried out in Japan, HPV/EBV coinfection was found in 1% of patients with HNC and in 10 % of patients with nasopharyngeal carcinoma (NPC) [31]. The coinfection with EBV and HPV in HNC is the most often found in NPC—it is more common in Asian than Caucasian populations and is most frequently reported in patients from endemic regions such as China, Iran, and Morocco [32,33,34]. Oropharyngeal tumors were also hypothesized to be associated with both HPV and EBV, since coinfected cells might have a higher tumorigenic potential than normal cells [27]. However, it is not clear which infection, HPV or EBV, is the first one [35].

Some researchers suggest that coinfections with high-risk HPV and EBV are important factors in initiation of carcinogenesis in human oral epithelial cells [6]. Both HPV and EBV have tropism for epithelial cells as they infect and replicate in the epithelium of the upper respiratory system and upper digestive tract. Several studies documented that oncoproteins of both viruses (LMP-1, LMP-2, E6 and E7) can cooperate and may induce oral epithelial cell transformation [6,11]. Guidry and Scott [28] suggested that HPV/EBV coinfection increases EBV persistence, either through latency or enhanced viral replication and by extending expression of HPV oncogenes. Makielski et al. [7] found that HPV18 promotes lytic reactivation of EBV in differentiating epithelial cells. The authors indicated that infection with HPV in the oral cavity may increase the capacity of epithelial cells to support the EBV life cycle, which could in turn contribute to pathogenesis mediated by EBV. Moreover, they stated that E6 and E7 HPV oncogenes are necessary and sufficient to promote EBV lytic reactivation mediated by HPV in the oral epithelial cells. However, the mechanism in which E6 and E7 cause EBV lytic reactivation remains to be elucidated. There is no experimental evidence that both viruses actually co-exist in the same cell, but coinfection may contribute to HNC in several ways. The authors reported that HPV stabilizes EBV in epithelial cells in the oral cavity, and that increased EBV persistence contributes to the likelihood of malignant transformation in this tissue. HPV, due to promotion of EBV lytic cycle, may increase viral yield, which can induce proliferation of neighboring cells [7].

It has been suggested that malignancies directly connected with several oncogenic viruses may be a result of prolonged latency following a chronic infection [11]. The mechanisms of the infection are complex and not fully understood. It is only in rare cases that infected individuals develop invasive cancer, therefore, it was stated that pathogenic infections are necessary but not sufficient for cancer initiation or progression [1,5,11,19,28]. It was documented that in chronic inflammation, epithelial and inflammatory cells generate ROS and reactive nitric species (RNS), and release growth factors and cytokines, which may cause DNA damage and alterations in critical pathways leading to cancer development or progression [36].

As several studies revealed, both EBV and HPV can act via ROS-based mechanisms. Long-term expression of the EBNA1 causes increased ROS and a nicotinamide adenine dinucleotide phosphate (NADPH) oxidase level [13,37]. According to some researchers, ROS formation may be induced by LMP1 [15]. Exposure of HPV16-infected cells to ROS results in an increased level of E6 and E7 as well as increased DNA damage [38,39].

Decreased antioxidant defense with increased oxidative stress was reported in patients with oral cavity cancer [40]. According to some reports, a lowered level of antioxidant enzymes may be crucial in lesion progression and can lead to the development of oxidative stress [41,42]. Our present study revealed significantly decreased GPx and SOD activity in patients with oropharyngeal cancer with HPV/EBV coinfection in relation to a single viral infection. No study comparing the level of oxidative stress parameters in patients with HNC coinfected with various viruses is available; therefore, we cannot relate our outcomes to the results obtained by other researchers. However, interesting research involving oxidative stress and infectious agents was carried out on a human cell model by Marullo et al. [18]. The authors revealed that oxidative stress plays a key role in promoting genomic instability in HPV(+) HNC cells. Moreover, they observed a state of chronic oxidative stress in HPV(+) HNC cells and reported that expression of HPV oncoproteins E6 and E7 is sufficient to induce ROS generation via NOX2 which led to oxidative DNA damage.

Studies concerning oxidative stress and EBV documented that oxidative agents causing damage to DNA induce the expression of EBV lytic genes. Carcinogens and EBV lytic infection synergistically increase oxidative stress, which is an integral link between environmental factors and EBV-associated cancers [43].

As some studies revealed, decreased antioxidant enzyme activity may also be connected with an advanced stage of malignant lesion and with the lowering level of histological differentiation of HNC tumor [42,44]. Srivastava et al. [45] documented lower values of, e.g., SOD and GPx in oral cancer patients with stage II, III, and IV according to TNM classification. Our outcomes are consistent with these findings as with advanced tumor dimensions and lymph-node involvement, the values of oxidative stress parameters were decreased. We have also noted that in poorly-differentiated tumors, GPx and SOD activity was decreased compared with well-differentiated lesions. In another study available in the literature performed in HNC patients, poorly and moderately-differentiated tumors were identified more frequently with lower total antioxidant status (TAS). The authors also found increased damage of DNA in cancer patients and supposed that it may be connected with insufficient antioxidant capacity and excessive ROS generation which contributes to the pathogenesis of cancer in HNC patients [42].

A relationship was also reported between EBV and poor differentiation of the tumor tissue in OSCC [46]. In experiments carried out by other researchers, EBV infection was found to delay epithelial differentiation and increase the invasiveness of epithelial cells expressing HPV E6 and E7 oncogenes. As delayed differentiation and enhanced invasiveness were still stated in epithelial cells after EBV loss, it was suggested that EBV infection may lead to epigenetic reprogramming [47].

Our study is the first original observation that implicates HPV and EBV coinfection with selected parameters of oxidative stress in pharyngeal cancer in the Polish population. A limitation of our research is, however, the small size of the group with coinfection, which makes statistical data comparing the relationship between viral coinfection and histological grading or TN classification insufficiently strong. HPV and EBV are the most commonly transmitted human viruses, but the prevalence of EBV and HPV infection is much more frequent than the incidence of malignant lesions connected with these infections. Finding factors or processes which lead to malignant transformation may be helpful in predicting which individuals exposed to these ubiquitous viruses may develop malignant tumors. Moreover, biological markers associated with viral infections and/or oxidative stress might be useful in diagnosis and treatment of cancer connected with oncogenic viruses, e.g., the treatment of EBV-positive tumors may include the use of targeted inhibitors of signaling cascades. Similarly, disorders in antioxidant enzyme status may be considered a possible biomarker and therapeutic target in cancer treatment strategy. Therefore, further studies are necessary to elucidate the influence of viral interplay, including EBV lytic reactivation induced by HPV and/or oxidative stress on malignant transformation.

## 5. Conclusions

The present study revealed significantly decreased level of GPx and SOD activity in oropharyngeal cancer tissue with EBV/HPV coinfection compared with a single infection with EBV or HPV. In poorly-differentiated tumors, GPx and SOD activity was decreased compared with well-differentiated lesions. In cases with greater tumor dimensions (T3-T4) and lymph-node involvement (N3-N4), the level of antioxidant enzymes was lowered. Biological markers associated with viral infection and/or oxidative stress might be useful in diagnosis and treatment of cancer connected with oncogenic viruses.

## Figures and Tables

**Table 1:** Epidemiological and clinical characteristics of oropharyngeal cancer patients with human papilloma virus/Epstein–Barr virus (HPV/EBV) coinfection or with EBV(+) or HPV(+) single infection.

		EBV + HPV *n* = 30		EBV(+) *n* = 54		HPV(+) *n* = 44	
		*n*	%	*n*	%	*N*	%
**Sex**	**female**	3	10.0	6	11.1	5	11.4
	**male**	27	90.0	48	88.9	39	88.6
***p***				0.9817			
**Age**	**<50**	5	16.7	9	16.7	7	15.9
	**50–69**	18	60.0	34	63.0	28	63.6
	**≥70**	7	23.3	11	20.3	9	20.4
***p***				0.9975			
**Place of residence**	**Urban**	24	80.0	43	79.6	34	77.3
	**Rural**	6	20.0	11	20.4	10	22.7
***p***				0.9469			
**Smoking**	**Yes**	23	76.7	41	75.9	33	75.0
	**No**	7	23.3	13	24.1	11	25.0
***p***				0.9861			
**Alcohol abuse**	**Yes**	27	90.0	49	90.7	40	90.9
	**No**	3	10.0	5	9.3	4	9.1
***p***				0.9906			
**Histological grading**	**G1-G2**	13	43.3	23	42.6	19	43.2
	**G3**	17	56.7	31	57.4	25	56.8
***p***				0.9972			
**T stage**	**T1-T2**	22	73.3	39	72.2	32	72.7
	**T3-T4**	8	26.7	15	27.8	12	27.3
***p***				0.9939			
**N stage**	**N1-N2**	23	76.7	41	75.9	33	75.0
	**N3-N4**	7	23.3	13	24.1	11	25.0
***p***				0.9860			
**M**	**M0**	30	100.0	54	100.0	44	100.0
Pearson’s Chi-square test							

**Table 2:** Activity of glutathione peroxidase (GPx) (U/mg of protein) and superoxide dismutase (SOD) (U/100 mg of protein) in tissue of EBV(+), HPV(+) and EBV/HPV coinfected patients with oropharyngeal cancer.

	***n***	**GPx**					**Kruskal–Wallis Test**	
		**M**	**SD**	**Min**	**Max**	**Me**	**H**	***p***
**EBV+**	54	4.47	2.11	2.05	9.55	4.73	8.169148	0.0168 *
**HPV+**	44	4.99	1.89	2.37	9.55	4.91		
**EBV+/HPV+**	30	3.75	2.09	1.22	9.55	3.32		
	***n***	**SOD**					**Kruskal–Wallis Test**	
		**M**	**SD**	**Min**	**Max**	**Me**	**H**	***p***
**EBV+**	54	11.06	1.59	9.11	13.91	10.83	13.37263	0.0012 *
**HPV+**	44	11.49	1.44	9.25	13.91	11.97		
**EBV+/HPV+**	30	9.77	2.24	5.22	12.44	9.81		

* statistically significant.

**Table 3:** Comparison between tissue activity of GPx (U/mg of protein) and SOD (U/100 mg of protein) and G,T,N in EBV/HPV coinfection, EBV(+) and HPV(+) patients with oropharyngeal cancer.

	EBV + HPV		EBV		HPV	
	GPx	SOD	GPx	SOD	GPx	SOD
**G1-G2**	5.74 ± 1.50	11.88 ± 0.55	6.33 ± 1.51	12.66 ± 0.57	6.58 ± 1.55	12.83 ± 0.42
**G3**	2.23 ± 0.76	8.15 ± 1.58	3.10 ± 1.26	9.87 ± 0.90	3.77 ± 1.03	10.47 ± 1.04
***p* value**	<0.001 *	<0.001 *	<0.001 *	<0.001 *	<0.001 *	<0.001 *
**T1-T2**	4.54 ± 1.88	10.87 ± 1.35	5.34 ± 1.85	11.77 ± 1.29	5.79 ± 1.58	12.19 ± 0.99
**T3-T4**	1.58 ± 0.32	6.76 ± 1.09	2.22 ± 0.21	9.21 ± 0.12	2.85 ± 0.31	9.62 ± 0.28
***p* value**	<0.001 *	<0.001 *	<0.001 *	<0.001 *	<0.001 *	<0.001 *
**N1-N2**	4.43 ± 1.91	10.77 ± 1.39	5.21 ± 1.90	11.65 ± 1.35	5.71 ± 1.62	12.13 ± 1.05
**N3-N4**	1.52 ± 0.30	6.47 ± 0.80	2.16 ± 0.12	9.17 ± 0.07	2.82 ± 0.30	9.59 ± 0.27
***p* value**	<0.001 *	<0.001 *	<0.001 *	<0.001 *	<0.001 *	<0.001 *

* statistically significant, Mann–Whitney *U* Test.
